# Anticancer Activity of Periplanetasin-5, an Antimicrobial Peptide from the Cockroach *Periplaneta americana*

**DOI:** 10.4014/jmb.2104.04040

**Published:** 2021-08-12

**Authors:** In-Woo Kim, Ra-Yeong Choi, Joon Ha Lee, Minchul Seo, Hwa Jeong Lee, Mi-Ae Kim, Seong Hyun Kim, Iksoo Kim, Jae Sam Hwang

**Affiliations:** 1 Department of Agricultural Biology, National Institute of Agricultural Sciences, Rural Development Administration, Wanju 55365, Republic of Korea; 2 College of Agriculture and Life Sciences, Chonnam National University, Gwangju 61186, Republic of Korea

**Keywords:** Periplanetasin-5, antimicrobial peptide, *Periplaneta americana*, anticancer activity, apoptosis

## Abstract

Cockroaches live in places where various pathogens exist and thus are more likely to use antimicrobial compounds to defend against pathogen intrusions. We previously performed an in silico analysis of the *Periplaneta americana* transcriptome and detected periplanetasin-5 using an in silico antimicrobial peptide prediction method. In this study, we investigated whether periplanetasin-5 has anticancer activity against the human leukemia cell line K562. Cell growth and survival of K562 cells treated with periplanetasin-5 were decreased in a dose-dependent manner. By using flow cytometric analysis, acridine orange/ethidium bromide (AO/EB) staining and DNA fragmentation, we found that periplanetasin-5 induced apoptotic and necrotic cell death in leukemia cells. In addition, these events were associated with increased levels of the pro-apoptotic proteins Fas and cytochrome c and reduced levels of the anti-apoptotic protein Bcl-2. Periplanetasin-5 induces the cleavage of pro-caspase-9, pro-caspase-8, pro-caspase-3, and poly (ADP-ribose) polymerase (PARP). The above data suggest that periplanetasin-5 induces apoptosis via both the intrinsic and extrinsic pathways. Moreover, caspase-related apoptosis was further confirmed by using the caspase inhibitor carbobenzoxy-valyl-alanyl-aspartyl-[O-methyl]- fluoromethylketone (Z-VAD-FMK), which reversed the periplanetasin-5-induced reduction in cell viability. In conclusion, periplanetasin-5 caused apoptosis in leukemia cells, suggesting its potential utility as an anticancer therapeutic agent.

## Introduction

Despite recent advances in treatment strategies, cancer remains the leading cause of death worldwide. In addition, the incidence of many cancers, including skin, prostate, breast, and kidney cancers, continues to rise. Cancer deaths are forecasted to continue to rise in 2030, with an estimated 11.5 million deaths. Traditional cancer chemotherapy, radiotherapy, and surgical treatment are primarily focused on causing mass cell death without high specificity, and thus often cause serious side effects and toxicity in various body tissues. Therefore, the development of novel tumor-targeting treatments that can effectively and specifically kill tumor cells while reducing toxicity to normal tissues is urgently needed [1-3].

Antimicrobial peptides (AMPs) are ubiquitous in nature and have been identified in and isolated from a diversity of living organisms. AMPs that possess potent antimicrobial efficacy against bacteria, fungi, and even certain viruses play important roles in the host innate immunity defense mechanisms of most living organisms, including plants, insects, amphibians, and mammals. Insect AMPs are cationic and amphipathic and exhibit various lengths, sequences and structures, but most have relatively small molecular masses (below 5 kDa). Numerous antibiotic peptides have been studied from a variety of insects, amphibians, and mammals, including humans [4-8]. Various anticancer activity studies have also been conducted recently using AMPs. MSP-4 is a marine AMP and natural compound derived from Nile tilapia (*Oreochromis niloticus*). MSP-4 can help induce the apoptosis of MG63 cells through the Fas/Fas ligand (FasL)- and mitochondria-mediated pathways, suggesting that it may be used as an innovative alternative treatment against human osteosarcoma [9]. In addition, CM4, extracted from the hemolymph of the Chinese silkworm *Bombyx mori*, induced K562/MDR cell necrosis by causing direct membrane disruption and significantly decreased the level of p-glycoprotein in K562/MDR cells [10].

*Periplaneta americana* (Linnaeus) is the most common cockroach species in the world and has a very high fertility rate. Cockroaches are being used as models for research in entomology, neurobiology, cardiovascular disease, and for the study of blood clotting mechanisms and microbial diversity. In particular, research on the discovery of proteins associated with allergies has been conducted using cockroaches [11]. In recent years, studies on genome decoding and transcript detoxification using next-generation sequencing (NGS) systems have been carried out, and interest in the various genetic characteristics of cockroaches and their utilization in such experiments have been increasing [12, 13].

In a previous study, we performed an in silico analysis of the *Periplaneta americana* transcriptome. Using an in silico AMP prediction method, 86 AMP candidates were predicted from the transcriptome, and among them, periplanetasin-5 was experimentally validated for its antimicrobial activity against yeast and gram-positive and gram-negative bacteria using a radial diffusion assay. It displayed little or no cytotoxic effects in a hemolysis assay [13]. We hypothesized that periplanetasin-5 might also have a cytotoxic effect on cancer cells, similar to that of some other AMPs. Therefore, in this study, we investigated the anticancer activity of the periplanetasin-5 peptide in K562 leukemia cells.

## Materials and Methods

### Peptide Synthesis

Periplanetasin-5 was synthesized by solid-phase peptide synthesis (SPPS) protocol (Anygen Co., Ltd., Republic of Korea). The peptide was dissolved in 0.01% aqueous acetic acid and stored at -20°C until use.

### Cell Culture

K562 cells were cultured in Roswell Park Memorial Institute 1640 (RPMI-1640) medium containing 10% fetal bovine serum (FBS), 100 units/ml penicillin G, and 100 μg/ml streptomycin (Gibco, Thermo Fisher Scientific, Inc., USA). Cells were cultured at 37°C in a humidified incubator with 5% CO_2_.

### Cell Viability and Caspase Inhibition Assays

K562 cells were plated into 96-well tissue culture plates (2×10^4^ cells/well) and treated with various concentrations (0, 10, 30, 50, 70, and 90 μg/ml) of periplanetasin-5 for 24 h. Cell viability was examined using CellTiter 96 AQueous One Solution Cell Proliferation Assay Kit (Promega Corp., USA). Absorbance was measured by using a microplate reader (DTX 8800 Multimode Detector; Beckman Coulter, Inc., USA) at 590 nm. Reversion of periplanetasin-5-induced viability reduction (50 and 70 μg/ml) was assessed by treating cells with carbobenzoxy-valyl-alanyl-aspartyl-[O-methyl]-fluoromethylketone (Z-VAD-FMK) (Promega Corp.), a broad-spectrum caspase inhibitor, at the indicated concentrations (100 and 200 μM).

### Lactate Dehydrogenase (LDH) Release Assay

Lactate dehydrogenase (LDH) release was detected by a Cytotoxicity Detection Kit (Roche Applied Science, Germany) to quantitatively assess cell death. Briefly, cells were plated at a density of 1 × 10^4^ cells/well into 96-well tissue culture plates in RPMI-1640 medium supplemented with 1% FBS. The cells were treated with different concentrations of periplanetasin-5 (0, 10, 30, 50, and 70 μg/ml) for 24 h. Post treatment, 5 μl of lysis solution was added to the positive control and incubated for 15 min. Then, 100 μl reaction solution was added to each well, and the plates were incubated for 15 min. Optical density was measured at 490 nm using a microplate reader (DTX 8800 Multimode Detector) following addition of 50 μl stop solution. Cytotoxicity was calculated using the following equation: cytotoxicity (%) = (exp. value - low control) / (high control - low control) × 100.

### Acridine Orange/Ethidium Bromide (AO/EB) Staining

K562 cells were seeded into 6-well tissue culture plates at a density of 1 × 10^6^ cells/well, treated with periplanetasin-5 (0, 10, 30, 50, and 70 μg/ml) for 24 h, and washed with PBS. The cells were stained with acridine orange (AO, 3 μg/ml) and ethidium bromide (EB, 10 μg/ml) and then viewed under a fluorescence microscope (Leica Microsystems, Germany).

### Annexin V/Propidium Iodide (PI) Staining

At the end of the treatment, cells were collected and washed twice with ice phosphate-buffered saline (PBS) and once with 1× binding buffer (10 mM Hepes/NaOH [pH 7.4], 140 mM NaCl, and 2.5 mM CaCl_2_). The cells (1 × 10^6^ cells) were then resuspended in 100 μl of binding buffer and added with 5 μl of fluorescein isothiocyanate (FITC) Annexin V and propidium iodide (PI). Subsequently, the cells were gently shaken and incubated at room temperature for 15 min in dark, after which 400 μl of 1× binding buffer was added into the cells. Stained cells were observed using flow cytometry with CytoFLEX (Beckman Coulter, Inc.).

### DNA Fragmentation Assay

K562 cells were seeded into 6-well tissue culture plates at 1 × 10^6^ cells/well and treated with periplanetasin-5 (0, 10, 30, 50, and 70 μg/ml) for 24 h. Untreated cells were used as the control sample. Then, cells were collected, washed once with PBS, lysed in a solution containing 10 mM Tris-HCl (pH 7.4), 10 mM ethylene-diamine-tetraacetic acid (EDTA, pH 8.0), and 0.5% Triton X-100 on ice for 30 min, and centrifuged at 15,000 ×*g* for 5 min. Next, the supernatant was digested using 0.1 mg of RNase A/ml and 1 mg of proteinase K/ml for 1 h at 55°C in the presence of 1% sodium dodecyl sulfate (SDS). DNA was extracted from the digested supernatant using phenol and chloroform, precipitated in cold ethanol, and subjected to electrophoresis on 2% agarose gels containing EB. DNA fragments were detected by ultraviolet (UV) light.

### Immunoblot Analysis

Cells were harvested and then suspended in NP-40 lysis buffer (Honeywell Fluka, USA) including protease and phosphatase inhibitors (Roche Applied Science). The protein concentration was measured using the Bradford Protein Assay Kit (Bio-Rad Laboratories, Inc., USA). The proteins were separated using 12% sodium dodecylsulfate polyacrylamide gel electrophoresis (SDS-PAGE) and transferred onto polyvinylidene fluoride (PVDF) membranes (Bio-Rad Laboratories, Inc.). After that, the membranes were blocked with 5% skim milk for 1h, and then probed with appropriate primary antibodies overnight at 4°C. The membranes were washed three times with TBST (25 mM Tris buffer, 0.15 M NaCl, 0.05% Tween 20) and incubated with the corresponding horseradish peroxidase (HRP)-conjugated secondary antibody (Promega Corp.) for 3 h at room temperature. Finally, the membranes were washed three times with TBST and developed with an enhanced chemiluminescence (ECL) detection kit (Invitrogen; Thermo Fisher Scientific, Inc.). Primary antibodies against cleaved caspase-3, -8, -9, poly (ADP-ribose) polymerase (PARP), cytochrome c, Fas, Bax, and Bcl-2 were purchased from Cell Signaling Technology, Inc. (USA). The β-actin antibody was obtained from Sigma-Aldrich (USA).

### Statistical Analysis

Results were presented as mean ± SD of three independent measurements and analyzed using one-way ANOVA followed by Student’s t-test. Data were considered statistically significant at *p* < 0.05.

## Results

### Cell Viability and Cell Membrane Disruption in Periplanetasin-5

**Fig. 1:**
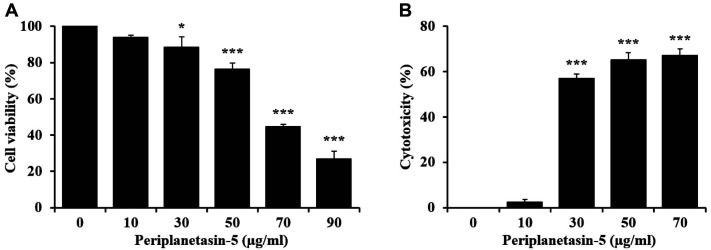
Cell viability and cell membrane disruption in periplanetasin-5-treated K562 cells. (**A**) Cell viability of cells treated with different concentrations (0, 10, 30, 50, 70, and 90 μg/ml) of periplanetasin-5. (**B**) Periplanetasin-5-induced cell membrane disruption based on the release of lactate dehydrogenase (LDH) in cells incubated with periplanetasin-5. Data are presented as the mean ± SD from three independent experiments. *p < 0.05 and ***p < 0.001 in comparison to untreated control.

We determined the effect of the synthetic peptide periplanetasin-5 on the cell viability and cytotoxic activity of human leukemia cells. As shown in Fig. 1A, periplanetasin-5 suppressed the growth of cancer cells in a dose-dependent manner. In particular, K562 cells treated with 90 μg/ml periplanetasin-5 showed a cell viability of approximately 30%. We determined the effect of periplanetasin-5 on loss of cell membrane integrity by detecting the release of LDH into media. As shown in Fig. 1B, the periplanetasin-5 caused excessive release of LDH compared to untreated K562 cells. Although the cytotoxicity did not change when the periplanetasin-5 was treated with doses up to 10 μg/ml, it increased rapidly when higher concentrations (30–70 μg/ml) were used. The cytotoxicity in K562 cells treated with 70 μg/ml periplanetasin-5 was over 60.0%, suggesting that the peptide has anticancer activity against human leukemia cells. These results indicated that the periplanetasin-5 could affect the survival of cancer cells by disrupting their membrane integrity.

### Determination of the Anticancer Activity of Periplanetasin-5 Using AO/EB Staining

**Fig. 2:**
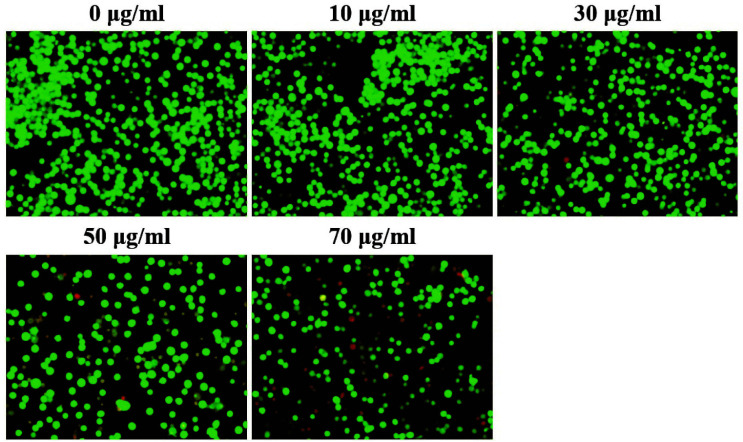
Determination of the anticancer activity of periplanetasin-5 using acridine orange/ethidium bromide (AO/EB) staining. Cells were observed under a fluorescence microscope (×200).

The anticancer activity of periplanetacin-5 was confirmed with the AO/EB staining method, which is commonly used for confirming cell death using real-time microscopy. In this method, living cells are stained with green fluorescence, while damaged/dead cells are stained with yellow or red fluorescence [14]. Our results demonstrated that cells treated with periplanetasin-5 at concentrations of 0–30 μg/ml showed a uniform cell shape, while many green-fluorescent cells were observed. However, the number of cells decreased at concentrations exceeding 50 μg/ml. Particularly, in the group treated with ≥ 70 μg/ml periplanetasin-5, the number of cells decreased and orange-red and red-fluorescent cells were observed (Fig. 2). Therefore, it was confirmed that periplanetasin-5 caused leukemia cell apoptosis and necrosis when cells were treated at doses ≥50μg/ml.

### Periplanetasin-5 Induced Apoptosis and Necrosis in Leukemia Cells

**Fig. 3:**
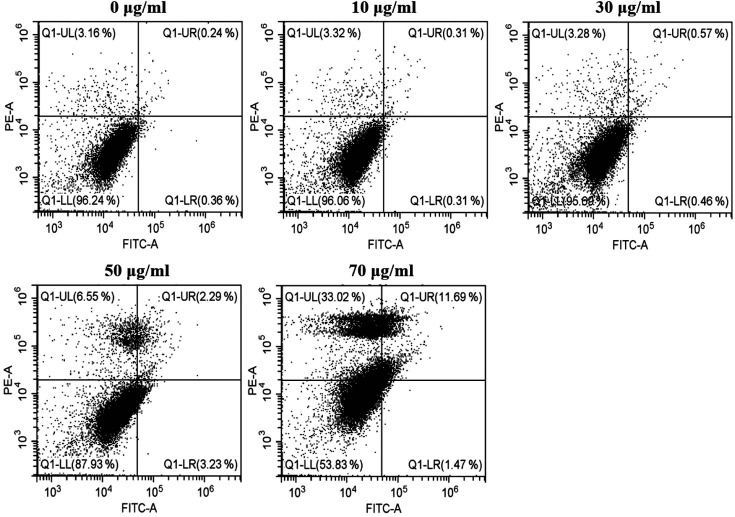
Periplanetasin-5 induces apoptotic and necrotic human leukemia cell death. K562 cells were treated with periplanetasin-5 (0, 10, 30, 50, and 70 μg/ml), and stained with FITC-conjugated Annexin V/propidium iodide. The lower left part represents viable cells, the lower right part represents early apoptotic cells, the upper left part represents necrotic cells, and the upper right part represents secondary necrotic and late apoptotic cells.

To demonstrate the periplanetasin-5-induced decrease of viability in K562 cells, we identified apoptotic and necrotic cells by characterizing their cytotoxic effects using Annexin V/PI staining and fluorescence microscopy or flow cytometry [15]. With 70 μg/ml periplanetasin-5 treatment, the cell population was distributed among late apoptotic and necrotic events. Fig. 3 shows that the apoptotic population of K562 cells increased by 3.23% after treatment with periplanetasin-5 at 50 μg/ml (early apoptosis) (Annexin V (+)/PI (-)). This indicated no significant change. However, the cells treated with periplanetasin-5 at 70 μg/ml increased proportions of Annexin V (+)/PI (+) (late apoptosis) and the Annexin V (-)/PI (+) (necrosis) cells. Late apoptosis increased by about 12%, and necrosis was increased by 33%. Thus, the periplanetasin-5 dose-dependently increased the Annexin V (+)/PI (+) and the Annexin V (-)/PI (+) populations suggesting that it induces both apoptosis and necrosis. These results demonstrated that the periplanetasin-5 at higher concentrations induced necrotic cell death.

### Periplanetasin-5 Induced DNA Fragmentation

**Fig. 4:**
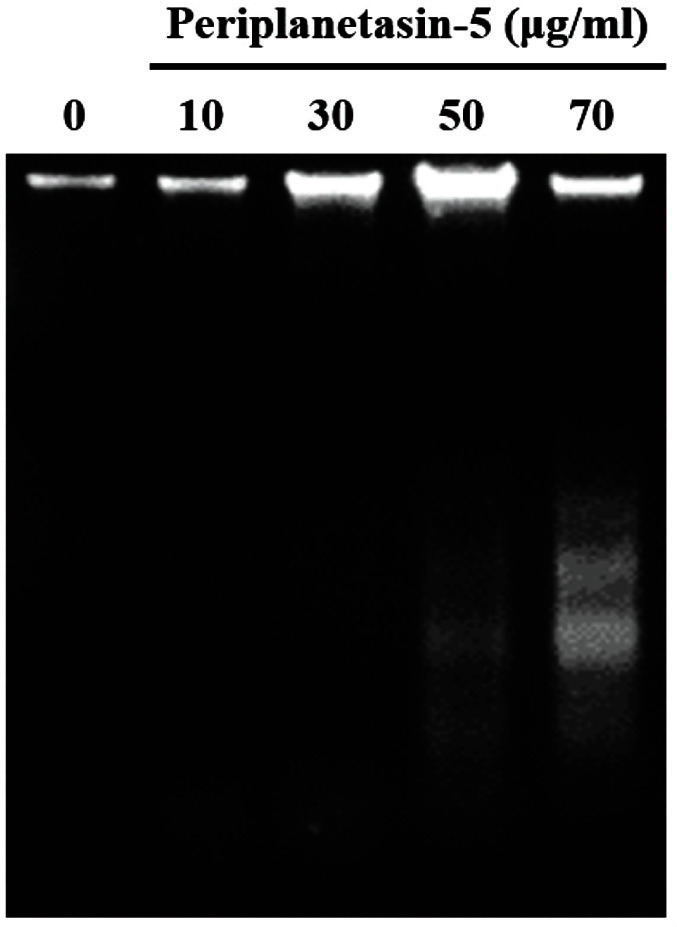
Periplanetasin-5 induces DNA fragmentation. DNA fragmentation analysis following K562 cell treatment with 0, 10, 30, 50, and 70 μg/ml of periplanetasin-5.

DNA fragmentation is a characteristic of apoptosis. In particular, the internucleosomal degradation of DNA is the final stage of apoptosis. DNA ladders that appear due to the activation of nuclear endonucleases are considered a hallmark in most cells undergoing apoptosis [16]. Therefore, DNA was collected directly from K562 cells treated with periplanetasin-5 to confirm fragmentation. As shown in Fig. 4, the chromosomal DNA of periplanetasin-5-treated K562 cells was fragmented in oligonucleosomal ladders. Particularly, DNA fragmentation was clearly observed at doses of 50–70 μg/ml. These results suggest that the periplanetasin-5-induced pro-apoptotic effects should contribute to a reduction in the viability of human leukemia K562 cells.

### Periplanetasin-5 Induced Apoptosis in Leukemia Cells via both the Intrinsic and Extrinsic Pathways

**Fig. 5:**
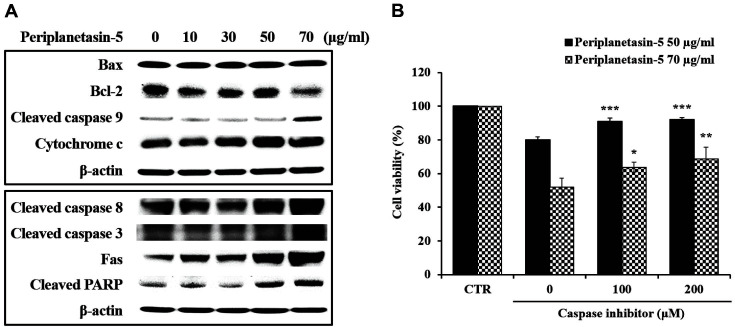
Periplanetasin-5 induces apoptosis in leukemia cells via both the intrinsic and extrinsic pathways. (**A**) Protein levels of caspase-3, -8, -9, PARP, cytochrome c, Fas, Bcl-2, Bax in K562 cells exposed to periplanetasin-5 (0, 10, 30, 50, and 70 μg/ml). (**B**) Cell viability of periplanetasin-5- treated (50 and 70 μg/ml) K562 cells following treatment with the caspase inhibitor Z-VAD-FMK (0, 100, and 200 μM). Data are presented as the mean ± SD from three independent experiments. *p < 0.05 and ***p < 0.001 in comparison to untreated control. CTR; control.

The mechanisms of apoptosis are categorized into intrinsic (mitochondria-mediated) and extrinsic (death receptor-mediated) pathways [17, 18]. Thus, we investigated the apoptotic pathways leading to periplanetasin-5-induced K562 cell death. As shown in Fig. 5A, expression of the cleaved form of caspase-3, -8, -9, and PARP proteins was increased by the periplanetasin-5 in a dose-dependent manner. With increasing concentrations of the periplanetasin-5, the expression of cytochrome c and Fas, which are apoptotic factors, increased, while the expression of the anti-apoptotic protein Bcl-2 decreased. However, the level of the pro-apoptotic protein Bax was not changed. These results confirmed that the periplanetasin-5 induced apoptosis via both the intrinsic and extrinsic pathways. Furthermore, the results suggest that periplanetasin-5-induced apoptosis was mediated through caspase activation in leukemia cells. To confirm this, cell viability after treatment with a caspase inhibitor was confirmed using the MTS assay. Z-VAD-FMK, a caspase inhibitor, inhibits caspase cleavage by binding to the active site of the caspase. As shown in Fig. 5B, following treatment with Z-VAD-FMK, the cell viability of K562 cells treated with 50 μg/ml periplanetasin-5 was restored to the cell viability levels of cells untreated with periplanetasin-5. However, the cell viability of K562 cells treated with 70 μg/ml of periplanetasin-5 showed a dose-dependent increase but was not completely recovered. These results suggest that early apoptosis was prevented by caspase inhibitor treatment in K562 cells treated with 50 μg/ml of periplanetasin-5. In the case of cells treated with 70 μg/ml periplanetasin-5, however, apoptosis and necrosis progressed simultaneously, suggesting that even if the caspase inhibitor sufficiently inhibited apoptosis, necrosis progressed and the survival rate did not increase greatly. Thus, our results demonstrated that the apoptosis of leukemia cells induced by periplanetacin-5 is dependent on the activation of caspase family proteins.

## Discussion

AMPs are small peptides essential for the congenital immune response of all species, indicating they are active against a wide range of pathogens [19]. Recently, anticancer activities have also been described for some of these peptides. These studies have demonstrated that treatment with Scolopendrasin VII from *Scolopendra subspinipes mutilans* and CopA3 from *Copris tripartitus* increased the release of LDH by leukemia and gastric cancer cells [20, 21]. In addition, PaDef, derived from avocados, was found to induce cell apoptosis in K562 cells related to the activation of caspase-8 [22]. Accordingly, it is possible that AMPs have great potential for use in cancer treatments in the future as a chemotherapy drug with a short time frame of interaction (reducing probability of resistance), low toxicity (reducing side effects), high specificity, good solubility, and good tumor penetration [23, 24]. Based on previous studies, we identified periplanetasin-5 from the cockroach’s transcriptome and found that it quickly decimated leukemia cells and caused the release of LDH.

Apoptosis, also known as programmed cell death, is a necessary process induced by various cell lesions that controls the number of normal cells by eliminating harmful cells. When apoptosis is induced in a cell, several morphological and biochemical changes take place, such as membrane blebbing, the condensation of the chromosomes by the decomposition of lamin-B1, the release of cytochrome c in mitochondria, and DNA fragmentation [25, 26]. AO/EB and Annexin V/PI staining results confirmed that periplanetasin-5 induces necrosis and apoptosis in leukemia cells. DNA fragmentation was clearly visualized at 50-70 μg/ml of periplanetasin-5 treatment, but not at higher doses. Thus, it is likely that low concentrations of periplanetasin-5 cause apoptosis, whereas higher concentrations lead to necrosis.

Apoptosis can happen through two different pathways: the intrinsic pathway and the extrinsic pathway. In both pathways, the process of apoptosis is performed by a caspase, an in-cell cysteine protease. The intrinsic pathway is caused by external stress, such as DNA damage. It is induced by the release of cytochrome c caused by the mitochondrial outer membrane permeabilization and by the activation of apoptosis protease-activating factor-1 (Apaf-1) and caspase-9. The extrinsic pathway leads to cell death as a result of the binding of a tumor necrosis factor, such as Fas, to the apoptosis receptor on the cell surface, activating caspase-8 through the Fas-associated death domain protein (FADD). Caspase-8 and -9, activated by these two pathways, activate the performance caspases (caspase-3 and -7) in the downstream pathway, leading to cell apoptosis by protein cleavage [26-28].

Periplanetasin-5 increased the levels of cytochrome c and cleaved caspase-9, important factors in the intrinsic pathway, and reduced the levels of Bcl-2. Furthermore, we confirmed an increase in the protein expression of Fas, cleaved caspase-3, -8, and PARP, which are the main routes of the extrinsic pathway. Thus, cell apoptosis in periplanetasin-5-treated cells is a result of intrinsic and extrinsic apoptotic pathway activation. According to Zhaoyu Jin’s study, despite the main role of caspase-8 in the extrinsic pathway, it is also known to be activated in response to some intrinsic cell death stimuli, causing it to facilitate caspase activation and accelerate death [28]. In other words, there is a crosstalk between the extrinsic and intrinsic apoptotic pathways mediated via caspase-8 cleavage of Bid (protein of the Bcl-2 family), which contributes to amplification of apoptotic signals through activation of mitochondrial pathway [29]. Therefore, although further research is required to elucidate this process, periplanetasin-5-induced apoptosis seems to be the result of the activation of intrinsic and extrinsic apoptosis pathways involving caspase-8 activation.

To determine the association between apoptosis and caspase activation in K562 leukemia cells, a caspase inhibitor was used to study changes in cell viability. Following treatment with the inhibitor, the survival rate was restored in cells treated with 50 μg/ml periplanetasin-5, but was not fully recovered in cells treated with 70 μg/ml periplanetasin-5. This confirmed that when lower concentrations of periplanetasin-5 were used, the apoptosis pathway progressed, while changes to the necrosis pathway occurred as the concentration increased. This is in line with the DNA fragmentation results.

In this report, we have shown that periplanetasin-5 is a novel and promising anticancer therapeutic agent candidate. We found that periplanetasin-5 has potential for therapeutic application in cancer and may be attributed to apoptosis and necrosis of leukemia cells.
